# The capillary adhesion technique: a versatile method for determining the liquid adhesion force and sample stiffness

**DOI:** 10.3762/bjnano.6.2

**Published:** 2015-01-02

**Authors:** Daniel Gandyra, Stefan Walheim, Stanislav Gorb, Wilhelm Barthlott, Thomas Schimmel

**Affiliations:** 1 Institute of Applied Physics and Center for Functional Nanostructures (CFN), Karlsruhe Institute of Technology (KIT), 76128 Karlsruhe, Germany; 2 Zoological Institute, University of Kiel, 24118 Kiel, Germany; 3 Nees Institute for Biodiversity of Plants, University of Bonn, 53115 Bonn, Germany,; 4 Institute of Nanotechnology (INT), Karlsruhe Institute of Technology (KIT), 76021 Karlsruhe, Germany

**Keywords:** adhesion, AFM cantilever, air layer, capillary forces, hairs, measurement, micromechanical systems, microstructures, Salvinia effect, *Salvinia molesta*, sensors, stiffness, superhydrophobic surfaces

## Abstract

We report a novel, practical technique for the concerted, simultaneous determination of both the adhesion force of a small structure or structural unit (e.g., an individual filament, hair, micromechanical component or microsensor) to a liquid and its elastic properties. The method involves the creation and development of a liquid meniscus upon touching a liquid surface with the structure, and the subsequent disruption of this liquid meniscus upon removal. The evaluation of the meniscus shape immediately before snap-off of the meniscus allows the quantitative determination of the liquid adhesion force. Concurrently, by measuring and evaluating the deformation of the structure under investigation, its elastic properties can be determined. The sensitivity of the method is remarkably high, practically limited by the resolution of the camera capturing the process. Adhesion forces down to 10 µN and spring constants up to 2 N/m were measured. Three exemplary applications of this method are demonstrated: (1) determination of the water adhesion force and the elasticity of individual hairs (trichomes) of the floating fern *Salvinia molesta*. (2) The investigation of human head hairs both with and without functional surface coatings (a topic of high relevance in the field of hair cosmetics) was performed. The method also resulted in the measurement of an elastic modulus (Young’s modulus) for individual hairs of 3.0 × 10^5^ N/cm^2^, which is within the typical range known for human hair. (3) Finally, the accuracy and validity of the capillary adhesion technique was proven by examining calibrated atomic force microscopy cantilevers, reproducing the spring constants calibrated using other methods.

## Introduction

Surface outgrowths such as hairs or trichomes, which widely occur on biological surfaces, sometimes have unique adhesive and elastic properties optimally adapted to specific functional requirements. As these are often mimicked in technical applications, their characterization is of great interest in a biomimetic approach. Prominent examples are the trichomes of the floating fern *Salvinia molesta*, which are responsible for the high air layer persistence of its leaves under water [1–3]. Artificial surfaces capable of retaining air under water have great potential in fluid transportation or as ship hull coatings [4–5] because of the significant drag reduction [6–12]. However, an essential requirement for the functionality of these surfaces is the persistence of the air layer [13–15]. As all of the highly engineered surfaces developed to date have failed in this respect [9–12], it is interesting to study the properties of the hairs of *Salvinia molesta* as a model for future developments. The key factors are the high water adhesion of the trichome tips (the “Salvinia effect”, [1]) and the high elasticity of the trichomes [2–3], which allows the pinning of the air–water interface by the trichome tips which hold it under perturbations without the loss of air by bubble formation.

Although the adhesive force of small elastic structures play a key role in understanding biological and biomimetic structures (as well as sensors, micromechanical or microfluidic systems), the precise, simultaneous measurement of both the elastic and the adhesive properties of small mechanical systems is not trivial. Here, we present a novel technique, the capillary adhesion technique (CAT), for the combined determination of the adhesion force of a single small structural entity to a liquid and its elastic properties. Capillary bridges were thoroughly studied with respect to kinetics and geometry dependence, in addition to the investigation and discussion of the contact angle and the related capillary length. The nucleation radius and growth of the liquid meniscus pulled from a flat surface were studied by Debregeas et al. [16]. The contact angle and contact angle hysteresis measurements on a curved surface (lens) pulling away from a meniscus were used to determine the advancing and receding contact angle of different solid materials [17] and the dynamics of this formation process [18]. Furthermore, the normal force of capillary bridges between solid objects was investigated [19].

Here, we create a meniscus from a flat, water surface until rupture occurs in order to determine the adhesion force using a simple energetic approach: the first derivative of the added surface energy of the meniscus with respect to the pulling direction. Using this method, we have investigated the hairs (trichomes) of *Salvinia molesta* and obtained precise values for the water adhesion force of the trichome tip and for the trichome elasticity. These data can now be used as a guideline for the design of biomimetic surfaces. To demonstrate the wide range of applications of this method, we also investigated human head hairs as a second example. Finally, calibrated atomic force microscopy (AFM) cantilevers were tested as a representative for an artificial micromechanical system, which at the same time allowed us to prove the validity and accuracy of the method.

## Results and Discussion

### The capillary adhesion technique (CAT)

**Figure 1:**
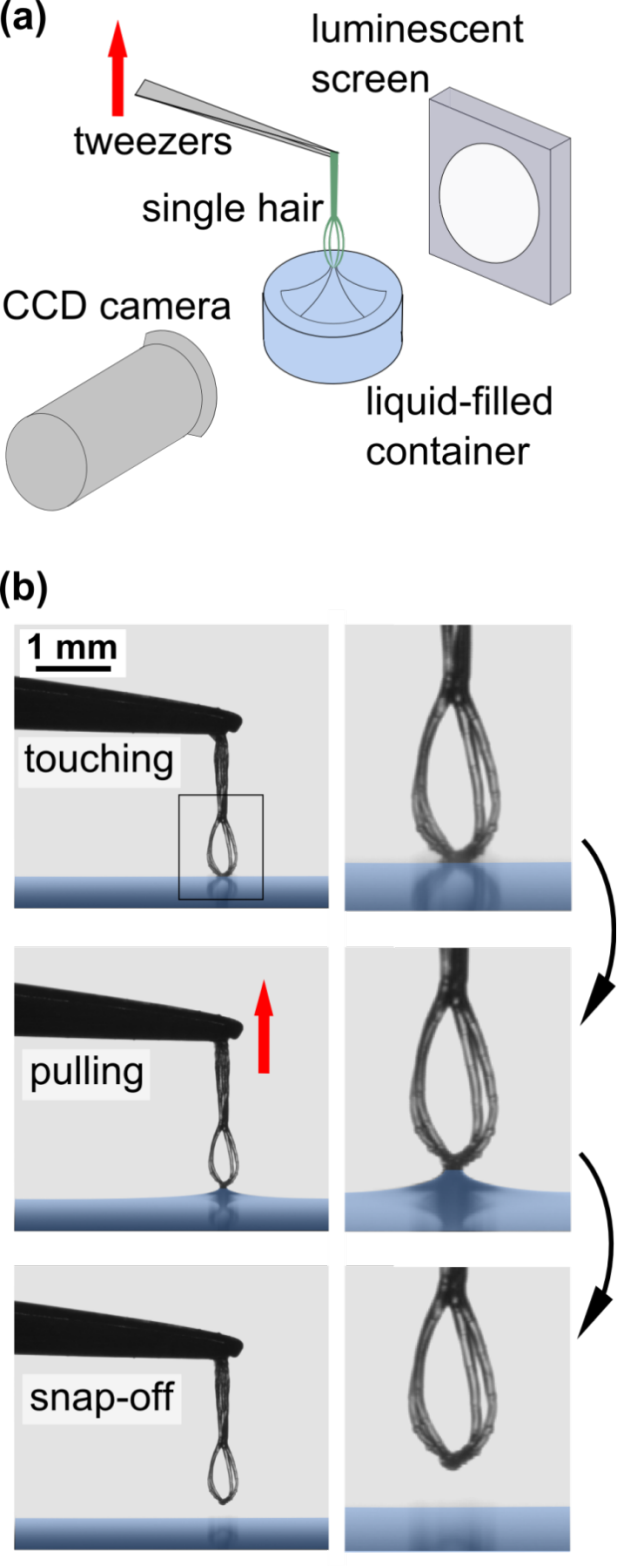
Description of the experimental method. (a) The experimental setup: A small elastic entity, in this case a hair (trichome) of *Salvinia molesta*, is placed between a luminescent screen and a CCD camera above a container filled with liquid. Using reverse action tweezers fixed on a stepper motor, the trichome is vertically descended onto the surface of the liquid (water). (b) After touching the liquid, the subsequent removal of the hair results in the formation of a meniscus. As the tip is pulled upwards, the meniscus eventually snaps off. The geometry of the mensicus immediately before snap-off (i.e., rupture of the meniscus) and the deformation of the trichome are recorded and evaluated.

The capillary adhesion technique differs substantially from common methods using various force sensors [20–23]. The principle is shown in Figure 1. A single small structure, here a trichome of the floating fern *Salvinia molesta*, is fixed at its base with reverse action tweezers. The tweezers are fixed on a stepper motor with the trichome tip facing downwards. Then, the tip is brought into contact with the surface of a liquid, here, water. When the trichome is pulled upwards, a meniscus is developed, which is eventually released when its tensile force exceeds the water adhesion force of the trichome tip. A constant pulling velocity of 120 µm/s was used. The pulling process is captured with a CCD camera, allowing the monitoring of both the meniscus shape and the deformation of the structure (here, the trichome) under investigation.

**Figure 2:**
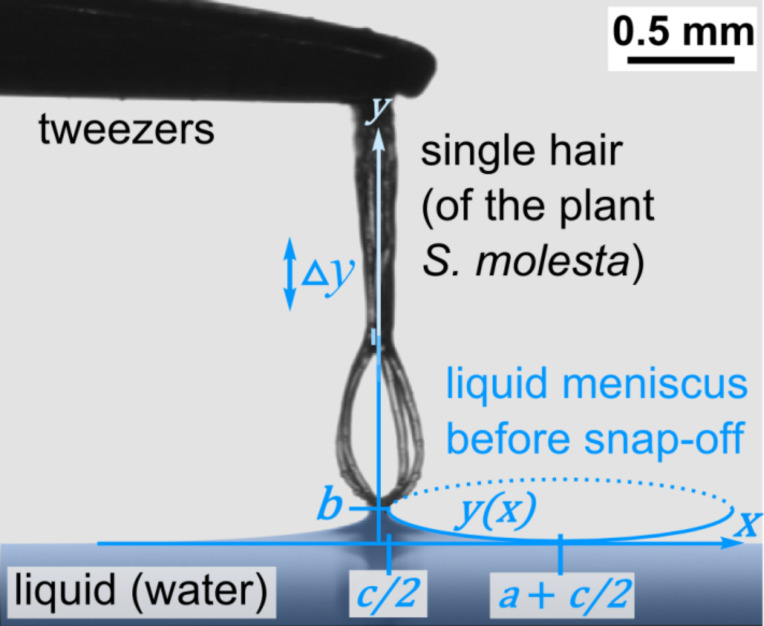
Meniscus immediately before snap-off. The profile can be fit by an elliptical function (Equation 1, *y*(*x*)) with fitting parameters *a*, *b*, *c* as shown above. From Equation 2, the surface area of the meniscus is derived, enabling calculation of the energy required to build the meniscus (Equation 3) and the maximum pulling force on the trichome (Equation 4) equivalent to the water adhesion force of the trichome tip. By evaluating the elongation of the trichome, Δ*y*, the elastic properties are determined.

Based on the image shortly before the meniscus snap-off, the profile of the meniscus can be fitted with an elliptic function:

[1]
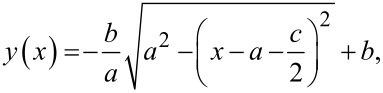


with parameters *a*, *b* and *c* as shown in Figure 2. The nature of the visual assessment of these parameters leads to a certain systematic error in the resulting adhesion force and elasticity values, which is essentially determined by the resolution of the CCD camera. The CCD camera resolution was 768 × 576 pixels. When the function *y*(*x*) is rotated around the *y*-axis, the rotationally symmetric shape of the meniscus in three dimensions can be modelled, which leads to the calculation of the resulting surface area [24] of the water meniscus as:

[2]
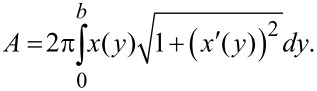


Wolfram Mathematica software was used for the calculations. To form the meniscus, beginning with the originally flat surface before capillary contact, a minimum energy of

[3]
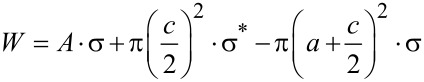


is required, which consists of the surface energy of the meniscus plus the interface energy of the tip–water contact area minus the surface energy of the original flat air–water interface before formation of the capillary contact. Here, σ = 0.07275 N/m [25] is the surface tension of the liquid (here, water), and σ* is the interface tension of the contact area. Although the value of σ* is unknown, it is not required for further calculations (see below). The force pulling at the trichome tip is equal to its water adhesion force and is given by:

[4]
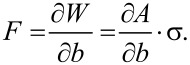


Based on the images before and after the meniscus snap-off, the elongation, Δ*y*, of the trichome in the direction of the force was observed. Assuming Hooke’s law, its spring constant is

[5]
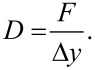


Likewise, other elastic constants such as Young’s modulus can be determined, as shown later in the section where human head hairs are examined. The contribution of the weight force of the water within the meniscus to the force pulling at the tip in contact with the water, is typically negligible. For the applications and examples shown in this work, the meniscus weight force was two orders of magnitude less than the contribution of the increasing liquid surface. This can be calculated using

[6]
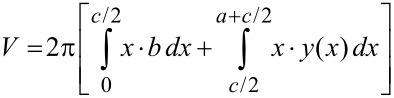


for the liquid volume within the meniscus, as given in [24], and thus

[7]
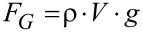


is the weight force, where ρ is the density of the liquid and *g* is the gravitational acceleration.

### Determining the adhesion force and elasticity of *Salvinia molesta* trichomes

The floating fern *Salvinia molesta* attracts considerable attention due to its ability to maintain a persistent layer of air on its leaves under water. This feature could be of high technological relevance, opening perspectives for completely new concepts for drag reduction of ships, for example, lubricating a ship hull with a permanent layer of air under water. As demonstrated, *Salvinia molesta* maintains this persistent air layer with a unique combination of hydrophobic hairs (trichomes) exhibiting hydrophilic, water-attracting tips (the Salvinia effect) [1]. The hydrophobic properties of the trichome surface prevent water from penetrating into the space between the multicellular trichomes, thus retaining a layer of air. The hydrophilic tips of the hairs, however, attract the water meniscus and adhere to the air–water interface, thus preventing the loss of air by formation of air bubbles.

In line with the method described above, 75 eggbeater-shaped trichomes of the floating fern *Salvinia molesta* were examined as taken from the central region of 15 adult leaves, 3 mm away from the edge, five from each of the leaves. This approach is explained by the fact that the trichomes at the edges exhibit different dimensions and shape. Our investigation resulted in an average value of the trichome tip adhesion force of *F* = (19.5 ± 0.3 ± 0.4) µN, where the statistical and systematic errors are noted, respectively. This high water adhesion force present at the tips of the eggbeater-shaped hairs is one key factor for the persistence of the air layer on the surface of submerged *Salvinia molesta* leaves due to the pinning of the air–water interface [1].

The elasticity of the trichomes was also studied, as there are indications that it may also play a key role in the air layer persistence. This elasticity may allow a moving air–water interface to be maintained by means of the hydrophilic pins, even under perturbations, without breaking water contact to the pins (which would lead to a loss of air by bubble formation) [1–3].

By applying CAT and investigating the 75 *Salvinia molesta* trichomes from the 15 different leaves mentioned above, we determined the average spring constant of the trichomes of *Salvinia molesta* to be *D*_pulling_ = (2.1 ± 0.2 ± 0.2) N/m, where the data denotes the average value, followed by the statistical and systematic errors, respectively. The data also support the assumption that the trichomes serve as soft springs (see above). The eggbeater shape of the trichomes is deemed to be ideal for this purpose. In fact, the experimental data show that the branched eggbeater-shaped part of the trichomes is responsible for the largest part of the length change of the trichomes as a response to an external force.

**Figure 3:**
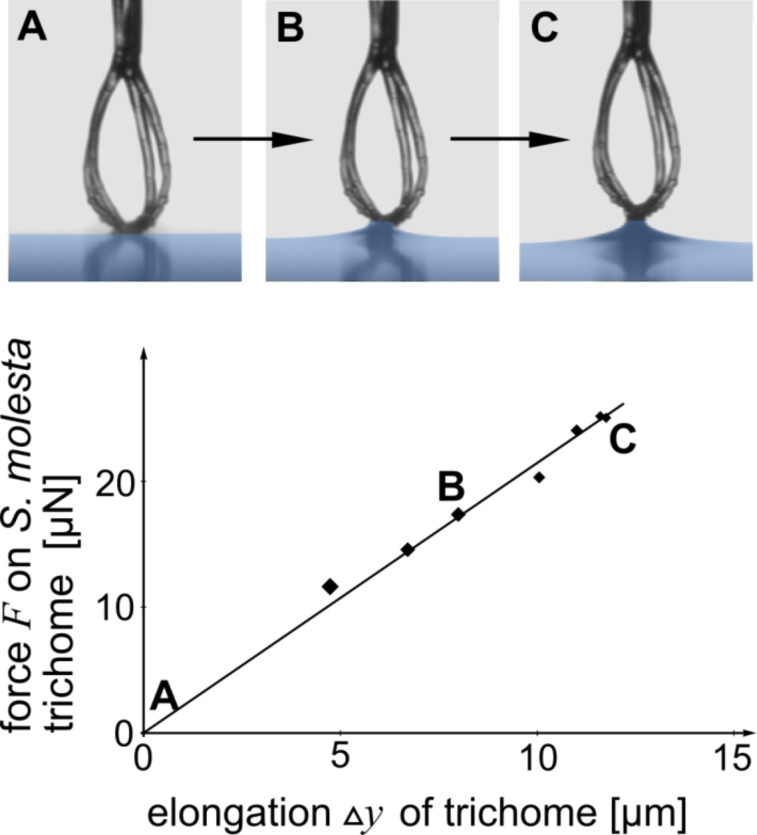
Force–elongation curve of a *Salvinia molesta* trichome. CAT allows force–elongation curves of small elastic structures to be measured based on the evaluation of the meniscus at different stages of the experiment. The linear fit to the data illustrates that the deformation follows Hooke’s law quite well: before snap-off, the elongation of the trichome shows a linear force dependence. The systematic error of the force *F* corresponds to half of the height of each measuring point, whereas the systematic error of Δ*y* was 3 µm in each case.

Determination of a spring constant according to Hooke´s law requires a linear elongation with increasing force, which is also proved by our method. In general, CAT allows the determination of the force–elongation curves of single structural entities. For this purpose, not only the image of the meniscus immediately before snap-off, but also other data taken between regarding the liquid surface–tip interaction and the snap-off are necessary to determine force and elongation. Figure 3 shows the results for a single *Salvinia molesta* trichome. As can be clearly seen, a linear function provides a very good approximation for the pulling force with respect to the trichome elongation, thus following Hooke’s law. This is valid over the whole range from a smaller force to the maximum force immediately before snap-off.

### Determining the water adhesion force, elasticity and Young’s modulus of human head hairs

As a second application example of CAT, the water adhesion force of human head hairs as well as their spring constant and Young’s modulus was studied by describing the elastic properties.

**Figure 4:**
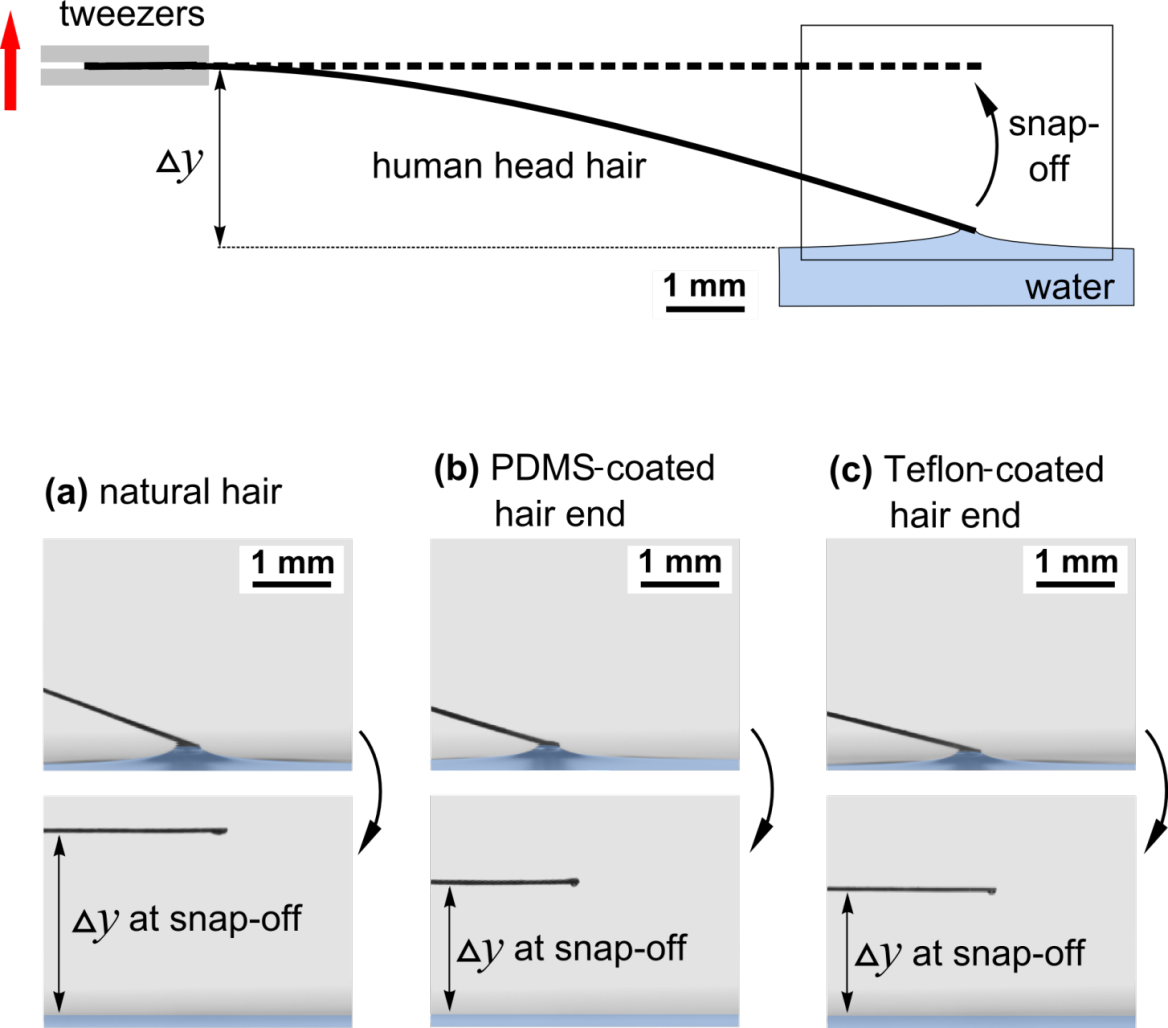
Examination of a human head hair. In this variation of the CAT, the hair is used as a bending spring. It is horizontally placed above the surface of the liquid in the tweezers and the bending before snap-off is measured with hair surfaces of different conditions (a) no coating (natural hair surface), (b) hair coated with teflon and (c) hair coated with silicone. Whereas the adhesion force depends on the coating, the elastic properties, such as the spring constant or Young’s modulus of the hair, remain unchanged by the coating.

For this purpose, hairs from the same person both in the natural state and with two different thin film surface coatings were used. The effect of the surface coating of human head hair on its adhesive properties is highly relevant, for example, in the field of hair cosmetics. We investigated natural hair, hair with silicone-coated ends (poly(dimethylsiloxane) (PDMS), Sylgard 184, Dow Corning, water contact angle approx. 110°) and hair with Teflon-coated ends (poly(1,1,2,2-tetrafluoroethylene) (PTFE), Teflon AF, Dupont, water contact angle approx. 120°). 15 hairs of each type were examined. They were fixed horizontally with the tweezers on a stepper motor, which served as a bending spring in contrast to the tension spring setup described in the previous section. The bending spring length was 1 cm. Figure 4 shows the experimental setup and images immediately before and after a typical meniscus snap-off event.

The results are summarized in Table 1. The water adhesion force of the ends of the hairs measured in their natural state was *F* = (44.7 ± 1.2 ± 0.6) µN where the data is followed by the statistical and systematic errors, respectively. This value is significantly higher than for the cases of the PDMS- or Teflon-coated hairs, that is, the adhesion force decreases when the hair ends are hydrophobically coated. In contrast, the bending spring constant remained the same (within the limits of accuracy of the measurement). Averaged over all 45 hairs, the bending spring constant was *D*_bending_ = (19.9 ± 0.9 ± 0.4) mN/m.

**Table 1:** The elastic and adhesive behavior of human head hairs with different coatings.^a^

	Natural hair	PDMS-coated hair end	Teflon-coated hair end

adhesion force (µN)	44.7 ± 1.2 ± 0.6	36.6 ± 1.0 ± 0.6	35.1 ± 1.3 ± 0.6
spring constant (mN/m)	19.1 ± 0.8 ± 0.3	20.6 ± 0.8 ± 0.4	20.1 ± 1.0 ± 0.4
Young’s modulus (× 10^5^ N/cm^2^)	2.96 ± 0.10 ± 0.20	3.10 ± 0.12 ± 0.23	3.07 ± 0.12 ± 0.22

^a^The data for each measurement is followed by the associated statistical and systematic error, respectively.

This version of CAT also allowed the measurement of Young’s modulus for the structures under investigation. In the case of an elastic bar acting as a bending spring (fixed at one end and pulled at the other end downwards), Young’s modulus is given by [26]

[8]
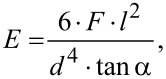


where the pulling force, *F*, is equivalent to the adhesion force, the hair length *l* = 1 cm, the hair diameter, *d* (individually and precisely determined using optical microscopy), and the angle α between the hair and water surface at the touching point immediately before snap-off, as extracted from the images shown in Figure 4. For the evaluation, the assumption of homogeneous and isotropic elastic properties was made. The resulting data are also shown in Table 1. For each type of hair coating (natural, PDMS- or Teflon-coated ends), the Young’s modulus was the same within the accuracy of the measurement. Its average value was *E* = (3.04 ± 0.11 ± 0.22) × 10^5^ N/cm^2^, with the statistical and systematic errors follow the data, respectively. The relative humidity during the measurements was roughly RH = 50% and the temperature was 22 °C.

Literature values citing Young’s modulus of human head hair are scarce. For example, in [27] a value of 3.89 × 10^5^ N/cm^2^ (RH = 60%) was indicated. In [28], a range between 1.5 and 4.6 × 10^5^ N/cm^2^ (RH = 65%) was indicated for 2–92 year-old humans, and in [29] a range of 1.23–4.10 × 10^5^ N/cm^2^ (RH = 30%) was given for 15–35 year-old humans. Thus, our results (3.04 × 10^5^ N/cm^2^, RH = 50%, hairs from a 29 year-old human) are in good agreement with these literature values, indicating that CAT yields reliable results.

### Validating the capillary adhesion technique using calibrated AFM cantilevers

**Figure 5:**
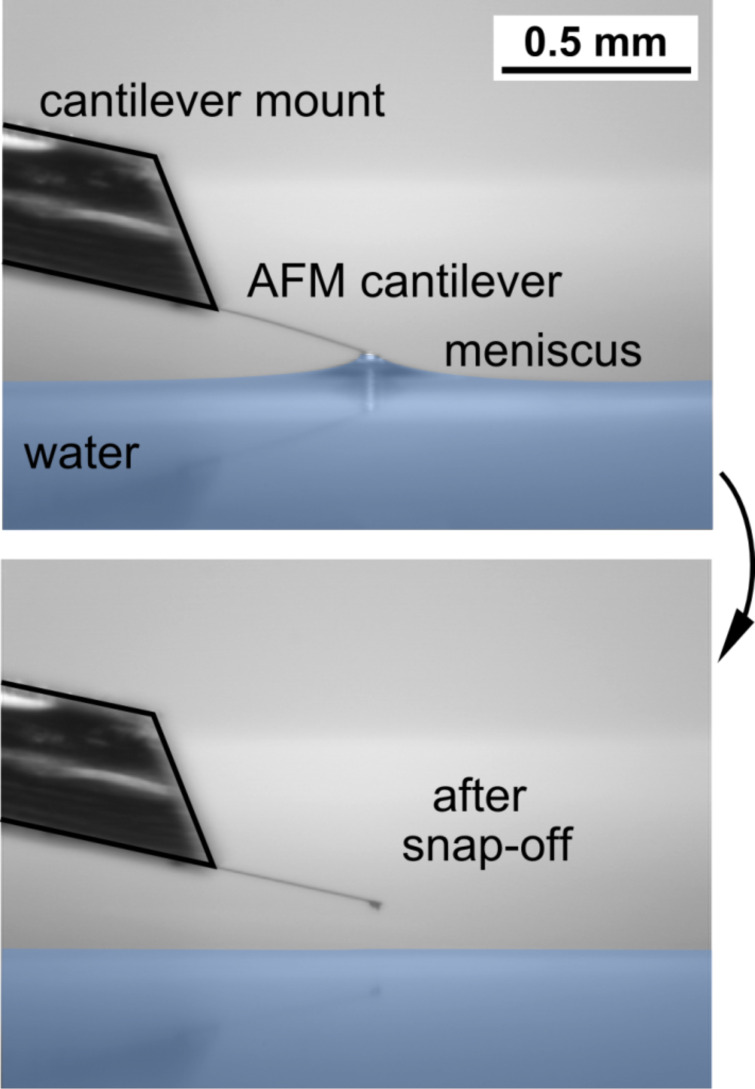
Proof of concept and accuracy of CAT, using specially calibrated AFM cantilevers. Determining the spring constant of the calibrated cantilever using the same settings as used for the individual hairs (see above, Figure 4) yields a spring constant in agreement with that given by the manufacturer.

A proof of the validity of the CAT method is given by examining a calibrated atomic force microscopy (AFM) cantilever. The cantilever was studied under the same conditions as the human head hairs (i.e., the chip on which the cantilever was attached was approximately horizontally fixed (11°) in the tweezers, see Figure 5). 15 independent measurements of the same calibrated cantilever were performed using the CAT, resulting in an average value for the water adhesion force of the cantilever tip of (11.8 ± 0.4 ± 0.3) µN and a spring constant of (0.195 ± 0.002 ± 0.011) N/m. This is in agreement with the spring constant of 0.18 N/m given by the manufacturer, which confirms the reliability of our method.

## Conclusion

To conclude, a versatile, novel technique was presented, which determines both the adhesion force of individual small mechanical entities to a liquid in addition to their elastic properties, both with remarkable sensitivity mostly limited by optical resolution limits. Using this method, the water adhesion force and the elastic spring constant of the tips of *Salvinia molesta* trichomes were determined as *F*_adhesion_ = (19.5 ± 0.3 ± 0.4) µN and *D* = (2.1 ± 0.2 ± 0.2) N/m, respectively, where the data is followed by the associated statistical and systematic error. As water adhesion and elasticity are key factors in order for *Salvinia molesta* leaves to maintain a persistent air layer under water, the resulting values can now be used as a basis for developing artificial air-retaining surfaces for technical applications based on a biomimetic approach. An example which further demonstrates the potential of this method was the investigation of the water adhesion force of natural and coated human head hairs, which is of high relevance for hair cosmetics, and the measurement of Young’s modulus of the hairs. The latter was determined to be 3.0 × 10^5^ N/cm^2^, which is in good agreement with values from literature. Finally, further proof of the validity of the capillary adhesion technique (CAT) was given by the measurement of the elastic force constant of 0.195 N/m for a calibrated atomic force microscopy cantilever.
